# Genomic profiles and clinical presentation of chordoma

**DOI:** 10.1186/s40478-024-01833-9

**Published:** 2024-08-12

**Authors:** Hela Koka, Weiyin Zhou, Mary L. McMaster, Jiwei Bai, Wen Luo, Alyssa Klein, Tongwu Zhang, Xing Hua, Xin Li, Difei Wang, Yujia Xiong, Kristine Jones, Aurelie Vogt, Belynda Hicks, Dilys Parry, Alisa M. Goldstein, Xiaohong R. Yang

**Affiliations:** 1 Division of Cancer Epidemiology and Genetics, National Cancer Institute, National Institutes of Health, Bethesda, MD USA; 2 Cancer Genomics Research Laboratory, Leidos Biomedical Research, Frederick National Laboratory for Cancer Research, Frederick, MD USA; 3 Department of Neurosurgery, Beijing Tiantan Hospital, Capital Medical University, Beijing, China; 4 Department of Pathology, West China Hospital, Sichuan University, Chengdu, China

**Keywords:** Chordoma, Chordoma sites, Treatment, Genomic landscape, Clinical outcome

## Abstract

Chordoma is a rare bone cancer with variable clinical outcomes. Here, we recruited 184 sporadic chordoma patients from the US and Canada and collected their clinical and treatment data. The average age at diagnosis was 45.5 years (Range 5–78) and the chordoma site distribution was 49.2% clivus, 26.2% spinal, and 24.0% sacral. Most patients (97.5%) received surgery as the primary treatment, among whom 85.3% also received additional treatment. Except for the most prevalent cancers like prostate, lung, breast, and skin cancer, there was no discernible enrichment for any specific cancer type among patients or their family members. Among a subset of patients (N = 70) with tumor materials, we conducted omics analyses and obtained targeted panel sequencing and SNP array genotyping data for 51 and 49 patients, respectively. The most recurrent somatic driver mutations included *PIK3CA* (12%), followed by chromatin remodeling genes *PBRM1* and *SETD2*. Amplification of the 6q27 region, containing the chordoma susceptibility gene *TBXT*, was detected in eight patients (16.3%). Clival patients appeared to be less likely to carry driver gene mutations, chromosome arm level deletion events (e.g., 5p, 5p, and 9p), or 6q27 amplification compared to sacral patients. After adjusting for age, sex, tumor site, and additional treatment, patients with somatic deletions of 14q (OR = 13.73, 95% CI 1.96–96.02, *P* = 0.008) and 18p (OR = 13.68, 95% CI 1.77–105.89, *P* = 0.012) were more likely to have persistent chordoma. The study highlights genomic heterogeneity in chordoma, potentially linked to location and clinical progression.

**Supplementary Information:**

The online version contains supplementary material available at 10.1186/s40478-024-01833-9.

## Introduction

Chordoma is a rare, slow-growing bone tumor (< 1 per 100,000) which is believed to originate from notochordal remnants that persist along the axial skeleton into adulthood. The usual sites of origin include the skull-base, mobile spine, and sacrum/coccyx. The incidence of chordoma increases with age and is very rare in young patients, particularly in the first decade of life [1]. Although chordomas are considered slow-growing, local recurrence is common.

Surgery is the mainstay of treatment for chordoma with radiation therapy as an optional adjuvant treatment, often depending on the location and extent of the tumor. Proton radiation therapy is regularly considered the best radiation treatment option, but its availability continues to be restricted [2]. There is presently inadequate clinical guidance on patient stratification regarding post-surgical treatment and treatment options for chordoma patients, particularly those with advanced disease, remain limited.

A better understanding of the molecular processes involved in chordoma tumorigenesis could lead to better-tailored treatments as well as improvements in prognostic prediction tools. We previously conducted whole genome sequencing (WGS) and RNA sequencing (RNA-Seq) in patients with skull-based chordoma from China and identified genomic alterations and molecular subtypes that were associated with patient clinical outcomes [3, 4]. Tumors in one expression subtype were more likely to have somatic mutations and reduced expression in chromatin remodeling genes, such as *PBRM1* and *SETD2*, whereas the other subtype was characterized by the upregulation of genes in epithelial–mesenchymal transition and Sonic Hedgehog pathways. However, genomic investigations of chordoma are limited particularly in datasets with well-annotated clinical data.

To gain insight into the predisposing and medical factors that might be related to chordoma prognosis, we evaluated clinical and questionnaire data collected from 184 chordoma patients recruited from the United States (US) and Canada. We conducted targeted panel sequencing and genome-wide SNP genotyping on a subset of 70 patients for whom tumor tissue and/or saliva samples were available.

## Methods

### Study population

The current study included 184 patients ranging from 5 to 78 years of age from the US and Canada, unselected for chordoma sites (Additional file 1). We posted the information about our study including the study background, goals, activities required to participate, and eligibility on the website of the NCI Division of Cancer Epidemiology and Genetics, The Chordoma Foundation, and The Chordoma Support Group. In addition, we also sent a letter describing our study and contact information to neurosurgeons and radiation therapists at medical centers in the US and Canada who specialize in the diagnosis and treatment of chordoma patients. Patients interested in participating contacted us and provided contact information for eligible subjects/parents. We then sent a letter to each eligible adult subject and the parents of each eligible minor subject that described the goals and methods of the study, including its voluntary nature, its major components (completing a self-administered personal and family medical history questionnaire, collecting a saliva sample using the Oragene DNA Self-Collection kit, and providing permission for NCI to obtain relevant medical records, pathology reports and pathology materials), and provided the toll-free telephone number that the recipients could call if they had questions about the study. Parents served as proxies for children who were 5–17 years old. All diagnoses of chordoma were confirmed by reviewing pathologic slides or reports, medical records, or death certificates. The study was approved by institutional review boards at the National Institutes of Health (all participants provided written informed consent).

### Genomic analyses

Formalin-fixed, paraffin-embedded (FFPE) tumor blocks or unstained sections were retrieved for 125 patients (Additional file 1). Thirty-one of them were removed due to insufficient tumor tissue in the block or few tissue sections available. Blocks/sections from 94 patients were processed to maximize tumor cells through macro-dissecting the tumor region and extracted for genomic DNA and total RNA. Among them, 24 samples had low DNA/RNA yield and/or quality, which led to 70 samples proceeding with downstream genomic analyses. Targeted sequencing was conducted using an AmpliSeq panel targeting 39 genes on the Ion Torrent S5 with an average coverage of 1158× per sample. The list of potential chordoma driver genes was compiled based on the work by Tarpey et al. (Additional file 2) [5]. After excluding samples with low sequencing quality, targeted sequencing data was available for 46 paired tumor and germline DNA (extracted from saliva) and 5 tumor-only samples. Torrent Variant Caller (TVC) was used for variant calling. Tumor/germline paired calls were made using the TVC algorithm implemented in Ion Reporter (V5.0.9). Variants were filtered out using the following criteria: *P*-value > 5.0E−6, flagged by the Confident Somatic Variants filter, allele frequency > 0.001 in the gnomAD database, variant allele fraction < 10% in tumor samples, and < 50 total reads. Tumor-only somatic variants were called using Torrent Variant Caller 5.0.2 and excluded if minimum allele fraction < 0.02, minimum coverage < 100, and minimum variant score < 6. Variants were annotated using snpEff, SnpSift and Annovar. We restricted our analysis to non-synonymous variants. Variant calls for targeted genes were checked manually through visual assessment using the Integrative Genomics Viewer (IGV). A genome-wide SNP array (GSAMD-24v1-0_20011747_A1, Illumina) was used to investigate somatic copy number alterations (SCNAs). After removing QC-failed tumor and germline samples, SCNA data was obtained from 49 tumors (42 tumor/germline pairs and 7 tumor-only samples).

The MoChA software was used to detect SCNAs in tumor/germline pairs and tumor-only samples [6]. MoChA uses hidden Markov models (HMM) to integrate Log R Ratio (LRR) and B Allele Frequency (BAF), leveraging haplotype information to detect subtle imbalances between maternal and paternal allelic fractions in a cell population. The Eagle software was used for phasing to infer haplotypes. LRR was used to determine the status of events (gain, loss, copy number neutral loss of heterozygosity [CNLOH]). All potential events were plotted and visualized, and false positive calls were excluded from the analysis based on the manual review of each plot.

RNA was quantitated using a ThermoScientific NanoDrop 2000 Spectrophotometer (cat. # ND-2000) and Agilent 4200 TapeStation. After assessing for low concentration or low percentages of RNA molecules > 300 nucleotides long (3%), the remaining samples were processed by the University of North Carolina Translational Genomics Laboratory using the Nanostring nCounter Platform. Samples were run on a custom code set that included gene sets selected from an independent RNA-Seq analysis [4]. Forty-eight samples passed the quality check and were included in the final analysis as previously described [4].

### Statistical analyses

The questionnaire asked about current health regarding chordoma with the following response choices: (1) having no chordoma anywhere in the body, (2) having chordoma at the original site but tumor was not growing, (3) having chordoma at the original site or elsewhere and tumor was growing, or (4) having chordoma metastasized at other sites. Follow-up data was available for only 53 patients, and therefore, to assess patient characteristics associated with clinical outcomes, we used the presence of chordoma, either due to incomplete dissection, recurrence, or metastasis, at the time of the questionnaire (i.e. combined groups 2, 3, and 4) as a surrogate prognostic measure or recurrence or death status when available. We examined this outcome in relation to clinical characteristics such as age at diagnosis, sex, and tumor site after adjustment for radiation therapy. When associating the chordoma outcome with genomic events, we were not able to adjust for additional treatment due to insufficient power. This proxy prognostic measure correlated well with the self-reported chordoma status from the follow-up data (Additional file 3). Since the majority of clival patients might not have complete surgical resection and the presence of chordoma is based on patients’ self-report, we also conducted a sensitivity analysis by combining groups 1 and 2 into one category (no growing chordoma) and groups 3 and 4 into another category (growing or metastatic chordoma). Fisher’s exact test was used to investigate differences of genomic and clinical characteristics between different groups. We utilized logistic regression models to assess the associations between clinical characteristics/outcome and genomic events with the adjustment for age at diagnosis, sex, and chordoma site. Among the 51 patients with targeted sequencing data, only three of them had received radiation treatment prior to surgery. Therefore, we did not adjust for pre-surgery radiation treatment in the regression model.

Unsupervised consensus clustering was conducted based on RNA Nanostring profiling and SNP array data to achieve gene expression and SCNA classification, respectively. We dichotomized the percent genome affected by SCNAs using the median value. Oncoplot was produced using the ComplexHeatmap package in R [7]. We utilized the Cancer Genome Interpreter (CGI) framework to identify potentially oncogenic mutations (https://www.cancergenomeinterpreter.org/home). All statistical tests in the present study were two-sided and performed using SAS version 9.4 (SAS Institute, Cary, NC, USA) or R version 4.3.1 (R Foundation for Statistical Computing, Vienna, Austria).

## Results

### Patient characteristics

Table 1 shows the clinical characteristics for 184 chordoma patients from the US and Canada included in this study. The average age at diagnosis was 45.5 years (range 5–78); the majority were females (56.3%) and non-Hispanic Whites (95.5%). The chordoma site distribution was 49.2% clivus, 26.2% spinal, and 24.0% sacral. Consistent with previous studies [8], we found that clival patients were associated with younger age at diagnosis (Mean = 41.8 years) than patients with sacral tumors (Mean = 52.3 years, *P* ≤ 0.0001). Twenty-one out of 155 patients (13.5%) reported having a history of other cancer either before (N = 12) or after chordoma diagnosis (N = 7). Only breast, prostate, and skin cancer were reported by more than one patient (Table 1). Eighty-eight patients (56.8%) reported having a family history of cancer among first-degree relatives, with breast, cervical, and skin cancer the most common cancer types among female relatives, and prostate, skin, and lung cancer the most prevalent among male relatives (Table 1). Two patients were subsequently found to have family members diagnosed with chordoma. All patients were alive at the time when the initial questionnaire was completed.

**Table 1:** Patient characteristics in 184 chordoma patients

Characteristic	N	%
Age at diagnosis (years)		
Mean, SD	45.5	15.4
< 18	9	4.95
18–30	22	12.1
30–50	68	37.4
≥ 50	83	45.6
Missing	2	
Sex		
Female	103	56.3
Male	80	43.7
Missing	1	
*Race/Ethnicity*		
Hispanic/Latino	7	4.5
Non-Hispanic white	150	95.5
Missing	27	
Chordoma site		
Clivus	90	49.2
Sacrum	48	26.2
Spine	44	24.0
Multiple	1	0.5
Missing	1	
*Personal history of additional cancer*		
No	134	86.5
Yes	21	13.5
Missing	29	
*Type of additional cancer*^*a*^		
Blood	1	4.8
Breast	2	9.5
Colon	1	4.8
Endometrial	1	4.8
Ovarian	1	4.8
Prostate	3	14.3
Skin	7	33.3
Solid pseudopapillary	1	4.8
Testicular	1	4.8
Thyroid	1	4.8
Unknown	2	9.5
Missing		
*Having additional cancer before chordoma diagnosis*		
No	7	36.8
Yes	12	63.2
Missing	2	
*Family history of cancer*^*b*^		
No	67	43.2
Yes	88	56.8
Missing	29	
*Type of cancer among FDR*^*c*^		
Bladder		3.1
Blood	5	3.8
Breast^d^	20	15.3
Cervix	8	6.1
Colon	6	4.6
Kidney	4	3.1
Liver	4	3.1
Lung	7	5.3
Lymphoma^e^	6	4.6
Prostate	19	14.5
Skin^f^	23	17.6
Other^g^	25	19.1
Missing		
*Number of additional treatments after surgery*		
1	71	55.9
2	25	19.7
3+	31	24.4
Missing	1	
*Chordoma outcome*^i^		
Not having chordoma anywhere in the body	76	49.7
Having chordoma at original site but tumor not growing	31	20.3
Having chordoma at original site or elsewhere and tumor was growing	26	17.0
Having chordoma spreading at other sites^j^	20	13.0
Missing	31	

^a^Self-reported data without confirmation

^b^Family history of cancer was defined as any other type of cancer in a first-degree relative

^c^FDR first-degree relative

^d^Includes one case of male breast cancer

^e^Includes Hodgkins and non-Hodgkins cases

^f^Includes basal cell (n = 6), melanoma (n = 14), Merkel cell (n = 1), and squamous cell (n = 2) carcinomas

^g^Includes abdomen (n = 2), appendix (n = 1), brain (n = 1), bone (n = 1), esophagus (n = 2), mouth (n = 2), nose (n = 1), ovary (n = 2), stomach (n = 1), pancreas (n = 2), penis (n = 1), testicle (n = 2), thyroid (n = 2), uterus (n = 3), unknown (n = 2) carcinomas

^i^Self-reported data among those with updated follow-up data

^j^Death cases are included in this category

Among 157 patients with treatment data available, the vast majority (97.5%) had surgery as their primary treatment after chordoma diagnosis, including 12 patients who received radiation prior to surgery (Table 1). The average time between chordoma diagnosis and the first surgery was 1.9 months, with almost all surgeries (97.3%) performed within a year. Most patients (85.3%) received additional treatment, including additional surgery (31.5%), proton radiation (42.5%), conventional radiation (24.4%), systemic (0.8%), and multi-modality (0.8%) treatment (Additional file 4). At the time of the administration of the initial questionnaire, which was on average 7.0 years after the first chordoma diagnosis, 76 patients reported not having chordoma anywhere, 31 had chordoma at the original site but the tumor was not growing, 26 had chordoma at the original site or elsewhere and the tumor was growing, and 20 had chordoma spreading to other sites (Table 1). Among 53 patients with a returned follow-up questionnaire 7–12 years after completing the initial questionnaire (on average 13.1 years since the first chordoma diagnosis), there were 12 reported deaths (Additional file 4), 9 patients (3 clivus, 4 spinal, and 2 sacral) reported a recurrence or relapse of chordoma (5 of them developed multiple recurrences/relapses), and 6 reported that the chordoma had metastasized to other organs (lung, abdomen, brain stem, and pancreas).

### Genomic profiles

**Fig. 1:**
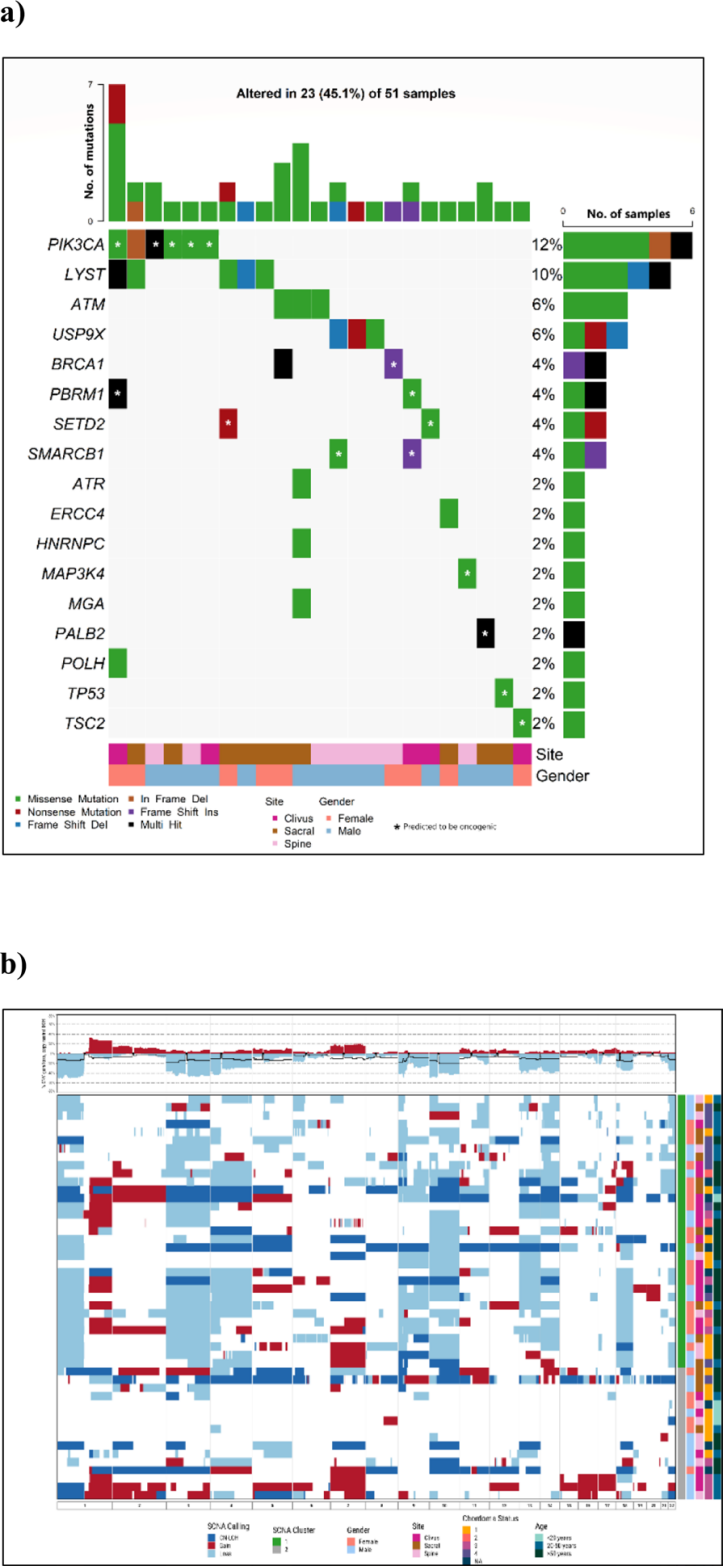
Genomic landscape of chordoma tumors: **A** Driver gene mutations; and **B** Somatic copy number alterations (Red: gain; Light blue: deletion; Dark blue: copy neutral LOH). Patients were clustered into two major groups. C1 (green): extensive SCNAs; C2 (gray): few or scattered SCNAs

After removing QC-failed samples at various steps, targeted sequencing, SNP array genotyping, and RNA profiling data were available for 51, 49, and 48 patients, respectively. Non-synonymous mutations were detected in 23 (45.1%) tumor samples, involving 17 potential chordoma driver genes (Fig. 1a). The most frequently mutated gene was *PIK3CA*, mutations in which were present in six (12%) patients, followed by *LYST* (10%), *ATM* (6%), and *USP9X* (6%). A notable observation was the seemingly mutually exclusive nature of mutations among *LYST*, *ATM* and *USP9X* as well as *PIK3CA*, *ATM*, and *USP9X*, although these are based on small numbers, within this patient cohort. Additionally, mutations within the chromatin remodeling genes *PBRM1*, *SETD2*, and *SMARCB1*, which have previously been recognized as frequently altered in chordoma [3, 5], were each identified in two patients. Using the CGI tool (https://www.cancergenomeinterpreter.org/home), we found that 53% of mutations shown in Fig. 1a were predicted to be driver rather than passenger mutations. Notably, almost all mutations in *PIK3CA* (except one), *PBRM1*, *SETD2*, *SMARCB1*, *TP53* and *MAP3K4* were predicted as driver mutations. One patient, who was a Hispanic female diagnosed with skull-base chordoma at the age of 26 and later developed metastases post-surgery and radiation therapy, exhibited both a missense mutation in *PBRM1* and a frameshift mutation in *SMARCB1,* both predicted as driver mutations. Consistent with previous findings that *TP53* mutations are rare in chordoma [3, 5], only one patient carried a *TP53* mutation, which was predicted as a driver mutation based on the CGI framework (Fig. 1a). In contrast, none of the mutations observed in *LYST*, *ATM*, *ATR*, and *USP9X* were predicted as driver mutations (Fig. 1a).

Somatic SCNAs were identified in the majority of tumors, with the percentage of genome affected by SCNAs ranging from 1 to 70% (mean = 30% across all patients). Frequent chromosome-level or arm-level SCNAs (i.e., 90% of the p or q arm of the chromosome covered by SCNAs) included gains of chromosomes 1q, 2, and 7, and deletions of 1p, 3, 4, 9p, 9q, 10, 13q, 14q, 18, and 22 (Fig. 1b). Clustering analysis revealed two distinct groups of patients based on SCNA events. The first group (C1, N = 33) demonstrated extensive SCNAs, while the tumors in the second group (C2, N = 16) had either few SCNAs or scattered events such as copy neutral loss of heterozygosity (LOH) events and copy number gains (Fig. 1b). Amplification of the 6q27 region, which harbors the chordoma susceptibility gene *TBXT*, was detected in eight patients (16.3%), six of whom exhibited high copy number gains.

We utilized a NanoString panel, comprising 21 of the most differentially expressed genes identified through an RNA-Seq analysis of skull-base chordoma samples [4], to perform molecular classification within our cohort. We observed two primary groups in 48 tumors, with tumors in one of the clusters (NCC2) showing upregulation of most examined genes, as previously described in Fig. 1 of our previously published work by Bai et al. [4].

### Genomic profiles in relation to clinical characteristics and outcomes

**Fig. 2:**
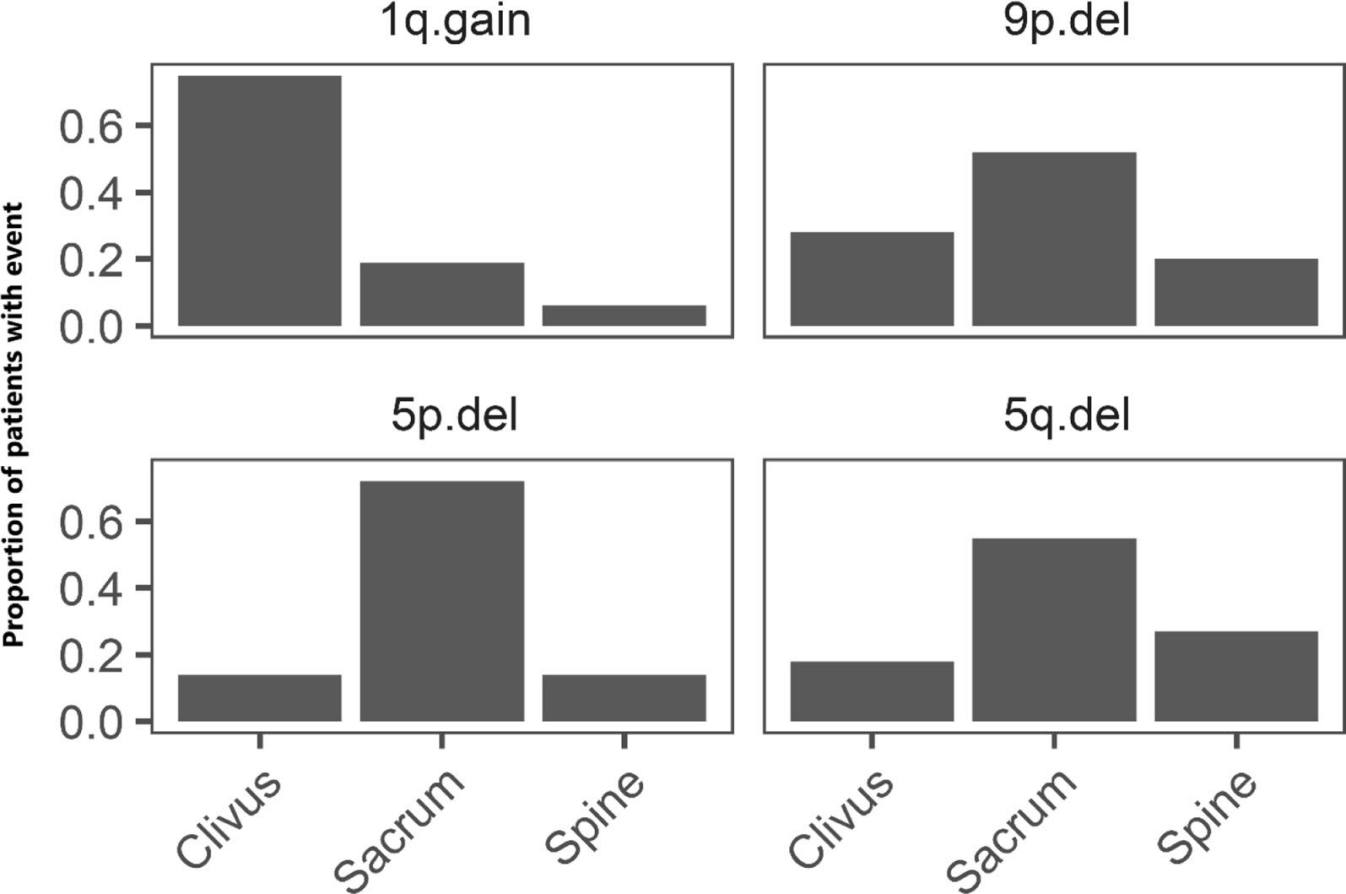
Distributions of genomic features by tumor location (N_Clivus_ = 21), (N_Sacrum_ = 14), (N_Spine_ = 14)

Among 63 distinct patients with any genomic data, the chordoma site distribution was 42.8% clivus, 28.6% spinal, and 28.6% sacral. The self-reported chordoma status distribution was: having no chordoma present anywhere (N = 24, 49.0%), having chordoma at the original site but the tumor was not growing (N = 5, 10.2%), having chordoma at the original site or elsewhere and the tumor was growing (N = 10, 20.4%), or having chordoma spreading to other sites (N = 10, 20.4%). Older patients (≥ 50 years) were more likely to have a higher percent of the genome affected by SCNA events (PGA, *P* = 0.005), particularly deletion events on chromosomes 1p, 3p, 4p, 4q, 10p, 10q, and 18q (Table 2). Interestingly, deletions of chromosome 4p were more prevalent among females than males (*P* = 0.004), while 7p gain was more common among males than females (*P* = 0.029, Table 2). Among 49 patients with both genomic and clinical data, 21 had clival, 15 had sacral, and 13 had spinal chordoma. After adjustment for age and sex, sacral tumors (OR = 5.76, *P* = 0.020) were more likely to harbor driver gene mutations compared to clival tumors. Deletions of chromosomes 5p (*P* = 0.033), 5q (*P* = 0.026) and 9p (*P* = 0.004) were more prevalent in sacral tumors compared to clival tumors, whereas chromosome 1q gain (*P* = 0.013) was more likely to be present in clival tumors (Table 2, Fig. 2). Among the tumors with *TBXT* amplification, two were clival, one was spinal, and the remaining five were sacral and from female patients. RNA expression subtype did not appear to be associated with age, sex, or chordoma site (Table 2). The individual diagnosed with a poorly differentiated chordoma had SNP array data exclusively accessible and was categorized into cluster C2 according to SCNA events observed in his tumor.

**Table 2:** Associations of genomic features with clinical characteristics and outcome in 49 patients

	Age at diagnosis (years)		Gender		Chordoma site				Chordoma presence^a^	
	≥ 50 vs. < 50		Male vs. Female		Sacrum vs. Clivus		Spine vs. Clivus		Yes vs. No	
	OR^b^ (95% CI)	*P*^b^	OR^b^ (95% CI)	*P*^b^	OR^b^ (95% CI)	*P*^b^	OR^b^ (95% CI)	*P*^b^	OR^a^ (95% CI)	*P*^a^
Deletion event										
1p.del	**6.03 (1.64–22.16)**	**0.007**	1.01 (0.27–3.74)	0.991	0.76 (0.16–3.63)	0.730	1.02 (0.21–4.95)	0.977	2.28 (0.40–13.09)	0.354
3p.del	**4.47 (1.21–16.52)**	**0.025**	0.83 (0.23–3.04)	0.775	0.50 (0.10–2.43)	0.389	0.34 (0.07–1.64)	0.180	4.53 (0.88–23.36)	0.071
3q.del	2.12 (0.61–7.32)	0.237	1.82 (0.49–6.72)	0.368	2.05 (0.42–10.11)	0.378	0.36 (0.08–1.59)	0.178	4.06 (0.78–21.16)	0.096
4p.del	**8.74 (1.56–48.97)**	**0.014**	**0.08 (0.02–0.44)**	**0.004**	2.07 (0.36–11.78)	0.413	0.47 (0.08–2.81)	0.407	**13.28 (1.11–158.60)**	**0.041**
4q.del	**3.62 (1.00–13.10)**	**0.050**	0.54 (0.15–1.90)	0.337	1.85 (0.43–7.89)	0.408	1.02 (0.22–4.69)	0.984	1.19 (0.26–5.46)	0.820
5p.del	0.63 (0.10–4.11)	0.630	0.35 (0.05–2.36)	0.281	**13.02 (1.23–138.14)**	**0.033**	2.10 (0.11–39.32)	0.620	2.13 (0.19–23.65)	0.537
5q.del	0.53 (0.12–2.40)	0.410	1.36 (0.30–6.13)	0.689	**8.46 (1.29–55.54)**	**0.026**	2.51 (0.34–18.26)	0.365	1.21 (0.21–7.05)	0.836
7p.del	4.53 (0.48–43.23)	0.189	0.90 (0.14–5.69)	0.909	0.36 (0.03–4.08)	0.409	0.97 (0.12–7.80)	0.979	0.17 (0.02–1.70)	0.130
9p.del	1.08 (0.28–4.19)	0.908	0.57 (0.14–2.28)	0.426	**26.82 (2.79–257.47)**	**0.004**	1.31 (0.29–5.84)	0.725	2.09 (0.35–12.51)	0.421
9q.del	0.79 (0.20–3.07)	0.727	0.55 (0.14–2.13)	0.388	1.58 (0.38–6.65)	0.531	0.18 (0.2–1.72)	0.136	1.09 (0.20–5.99)	0.920
10p.del	**4.10 (1.14–14.81)**	**0.031**	2.54 (0.72–8.98)	0.148	1.04 (0.24–4.59)	0.960	1.38 (0.30–6.23)	0.678	1.16 (0.25–5.31)	0.853
10q.del	**7.40 (1.85–29.62)**	**0.005**	1.40 (0.38–5.13)	0.612	0.52 (0.11–2.44)	0.405	0.64 (0.13–3.11)	0.577	2.50 (0.54–11.62)	0.242
13q.del	2.53 (0.73–8.78)	0.144	0.98 (0.29–3.32)	0.974	0.62 (0.15–2.55)	0.504	0.27 (0.06–1.26)	0.096	0.57 (0.14–2.38)	0.437
14q.del	1.43 (0.44–4.72)	0.555	0.65 (0.20–2.14)	0.481	1.54 (0.37–6.34)	0.552	0.56 (0.13–2.34)	0.423	**13.73 (1.96–96.02)**	**0.008**
18p.del	2.16 (0.63–7.42)	0.222	0.75 (0.22–2.52)	0.639	1.17 (0.29–4.71)	0.830	0.55 (0.12–2.49)	0.436	**13.68 (1.77–105.89)**	**0.012**
18q.del	**4.18 (1.07–16.39)**	**0.040**	0.55 (0.15–2.01)	0.363	2.31 (0.53–10.14)	0.268	0.89 (0.18–4.42)	0.882	2.75 (0.52–14.46)	0.233
22q.del	0.76 (0.23–2.50)	0.649	0.86 (0.26–2.83)	0.804	2.29 (0.56–9.35)	0.247	0.95 (0.22–4.06)	0.950	2.69 (0.58–12.52)	0.208
Gain event										
1q.gain	0.61 (0.15–2.42)	0.479	0.93 (0.23–3.70)	0.912	0.22 (0.05–1.05)	0.057	**0.06 (0.01–0.55)**	**0.013**	3.43 (0.59–19.93)	0.171
2p.gain	0.77 (0.12–4.80)	0.775	1.42 (0.23–8.69)	0.706	0.26 (0.03–2.59)	0.250			2.27 (0.20–26.22)	0.510
2q.gain	0.46 (0.06–3.42)	0.445	2.25 (0.31–16.27)	0.423	0.39 (0.04–4.15)	0.431			1.64 (0.13–20.97)	0.703
5p.gain	2.41 (0.33–17.55)	0.386	3.29 (0.43–25.23)	0.251			0.16 (0.01–1.77)	0.134	1.61 (0.12–21.98)	0.721
5q.gain	1.81 (0.27–12.23)	0.542	2.76 (0.41–18.53)	0.296	0.27 (0.03–3.00)	0.288	0.23 (0.02–2.58)	0.233	1.61 (0.12–21.98)	0.721
7p.gain	1.71 (0.31–9.29)	0.538	**11.93 (1.28–111.01)**	**0.029**	0.56 (0.07–4.54)	0.588	0.62 (0.10–3.92)	0.608	1.22 (0.21–7.12)	0.829
Genomic event										
Any driver gene mutation^c^	0.70 (0.20–2.54)	0.609	2.22 (0.60–8.15)	0.232	**5.76 (1.32–25.19)**	**0.020**	2.05 (0.45–9.37)	0.354	2.03 (0.51–8.17)	0.318
NCC2 vs. NCC1	2.26 (0.65–7.83)	0.199	1.37 (0.40–4.73)	0.615	1.30 (0.29–5.76)	0.729	1.20 (0.28–5.09)	0.805	1.43 (0.31–6.53)	0.647
SCNA (C1 VS. C2)	3.43 (0.91–12.92)	0.069	0.50 (0.13–1.86)	0.300	0.83 (0.17–4.08)	0.822	0.45 (0.10–2.15)	0.317	3.63 (0.70–18.93)	0.126
PGA^d^	**8.24 (1.91–35.48)**	**0.005**	0.32 (0.08–1.24)	0.099	0.52 (0.11–2.52)	0.776	0.41 (0.08–2.18)	0.454	1.96 (0.39–9.73)	0.413

^a^The outcome was dichotomized into presence of chordoma anywhere in the body versus no chordoma anywhere. The explanatory variable was each genomic feature. Models were adjusted for age, gender, and chordoma site

^b^Results are based on logistic regression models. The outcome was each genomic feature run separately. Models were adjusted for age, sex, and chordoma site

^c^Any mutation in ARID1A, ATM, BRCA2, ERCC4, LYST, MAP3K4, PALB2, PBRM1, PIK3CA, SETD2, SMARCB1, TP53, or TSC2

^d^PGA = Percent of the genome affected by somatic copy number alterations was dichotomized using the median value

OR = Odds ratio; Lower and Upper = 95% confidence interval

Compared to patients free of chordoma at the time of the questionnaire, patients with persistent chordoma or who died were more likely to have deletions of chromosome 4p (OR = 13.28, 95% CI 1.11–158.60, *P* = 0.041), 14q (OR = 13.73, 95% CI 1.96–96.02, *P* = 0.008), and 18p (OR = 13.68, 95% CI 1.77–105.89, *P* = 0.012, Table 2, Additional file 5). On the other hand, RNA expression subtype, SCNA cluster, PGA, or driver gene mutations were not significantly associated with the presence of chordoma (Table 2). We also conducted a sensitivity analysis comparing patients with chordoma that was growing or metastatic chordoma to those with either no chordoma present or no growing chordoma. The main findings did not differ significantly (Additional file 6).

## Discussion

The rarity of chordoma poses a significant challenge in comprehensively exploring its disease course, clinical behavior, and biology. In this study, we accrued 184 chordoma patients residing in the United States and Canada and collected information on personal and family history of cancer, treatment for chordoma, and chordoma status through questionnaires. The collection of tumor materials and saliva samples from a subset of these patients enabled us to perform molecular analyses, validating molecular markers previously identified in recent omics studies [3, 5]. Our genomic data confirmed previous findings that *PIK3CA* and chromatin remodeling genes were among the most frequently mutated genes in chordoma, while the potential driver role of *LYST* and *USP9X* were less certain. Results from our study also underscore the heterogeneous nature of chordoma, revealing variations based on tumor location and genomic features, which appear to be interrelated. This emphasizes the importance of integrating molecular markers into clinical management practices.

Most patients in this study underwent additional treatment following the initial tumor resection, with over half of patients having multiple additional treatments. At the time of questionnaire administration, conducted on average approximately 7 years post-initial chordoma diagnosis, nearly half of the patients exhibited chordoma persistence either at the original site or in other locations. This underscores the formidable challenge of achieving complete tumor resection, particularly evident in clival chordoma patients, who were more prone to needing additional treatment and having residual tumor at the original site. It is worth noting that advancements in surgical techniques may have contributed to improvements in recent years. Although clival patients are more likely to have chordoma persistence, metastases are known to occur more commonly among non-clival than clival patients [9]. Data from the current analysis also revealed a higher incidence of metastasis among sacral patients, although the difference did not reach statistical significance, possibly due to the small sample size. Further, we previously have shown that sacral patients in chordoma high-risk families were more likely to have larger tumors than clival patients [1]. While the larger tumor size and higher propensity for metastasis might be attributed to delayed diagnosis due to a lack of symptoms in the early stages, our genomic findings suggest that sacral tumors may exhibit a more aggressive biological behavior compared to clival tumors. We found that sacral patients were more inclined to harbor driver gene mutations, deletion events, such as the deletion of chromosome 9p containing the tumor suppressor gene *CDKN2A*, and *TBXT* amplifications. These insights shed light on the complexities of chordoma progression and underscore the importance of tailoring treatment strategies based on the specific characteristics of the tumor's location.

Researchers from the University of Pittsburgh previously reported an independent association between 1p36 and 9p21 deletions and shorter survival among clival patients [10]. They classified patients into three risk groups based on the combinations of these two markers. Similarly, our previous analysis of clival chordoma tumors using whole-genome sequencing revealed that deletions of 9p and 9q were linked to worse recurrence-free survival. However, we demonstrated that the combined alterations of *PBRM1* and 22q deletion were more significant in predicting prognosis [3]. In the current study, encompassing all tumor locations, we identified potential prognostic value in deletions of two additional chromosomal regions, 14q and 18p. The variability in these findings underscores the importance of validating these markers in studies with many patients using standardized assays. The panel of 1p36 and 9p21 markers has already been incorporated into some pathology laboratories. In our patient cohort, spanning nearly five decades of chordoma diagnoses, only the four most recently diagnosed patients were tested for this panel. Future studies are warranted to evaluate the predictive value of these genetic markers with more comprehensive data available. Additionally, there is a need to develop a more comprehensive scheme that incorporates both genetic and epigenetic markers, along with targets related to the tumor microenvironment, to refine prognostic classification.

Chordoma has been noted for its morphological resemblance to clear cell renal cell carcinoma (ccRCC) [11]. Our previous whole-genome sequencing (WGS) analysis revealed similar mutational signature profiles for chordoma and ccRCC [3]. However, none of the chordoma patients in our study exhibited ccRCC, and kidney cancer was not prevalent among their first-degree relatives. Moreover, apart from the most prevalent cancers like prostate, lung, breast, and skin cancer, there was no discernible enrichment for any specific cancer type among patients or their family members.

Our study is relatively small due to the challenges of researching rare cancers, which have limited our statistical power, especially in identifying associations with molecular data. While we invited all chordoma patients in the US and Canada to participate, our study population may not fully represent the general patient demographic. For instance, patients with more advanced disease might not have been included in the study since they may have been too ill or have died before they were enrolled into the study. The molecular analyses conducted are susceptible to selection bias, as they rely on high-quality tumor materials. Additionally, the variability in surgical and pathology reports is significant due to patients being diagnosed and treated at different times and in different hospitals. Information on immunohistochemical markers and histology types is also limited. Our study is primarily cross-sectional and questionnaire-based, with limited longitudinal follow-up data, which hinders our ability to assess survival associations. Lastly, our exploration of relationships between genomic and clinical features was largely exploratory, limited by the small number of patients with genomic data.

In conclusion, our study provides a comprehensive clinical and molecular landscape of chordoma across multiple sites. The identified heterogeneity in chordoma underscores the complexity of the disease. The insights gleaned from our research provide a deeper understanding of chordoma, laying the groundwork for the development of personalized management and treatment options.

### Supplementary Information

Additional file 1.

Additional file 2.

Additional file 3.

Additional file 4.

Additional file 5.

Additional file 6.

## Data Availability

The targeted sequencing and NanoString data for the European ancestry replication dataset have been deposited in dbGaP under accession # phs001280.v1.p1.
