# Supplementary figures and images for: Genomic profiles and clinical presentation of chordoma
Source: Acta Neuropathol Commun. 2024 Aug 12;12:129. doi: 10.1186/s40478-024-01833-9 (PMC11318126; doi:10.1186/s40478-024-01833-9)

**Additional file 1: Study design**


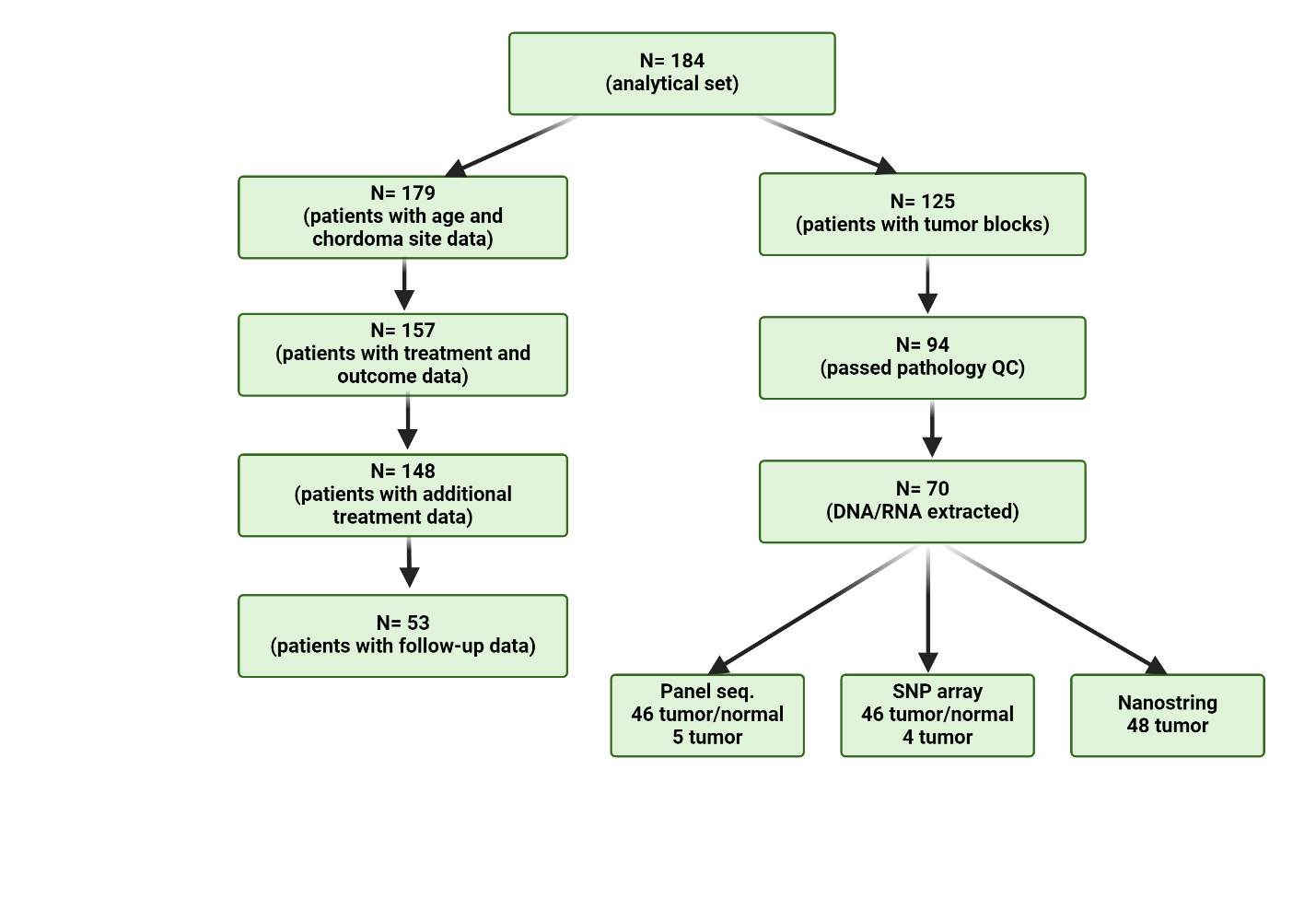

Supplement: Supplementary file 1 — Additional file 1. [file 40478_2024_1833_MOESM1_ESM.docx]
