# Supplementary material for: Genomic profiles and clinical presentation of chordoma
Source: Acta Neuropathol Commun. 2024 Aug 12;12:129. doi: 10.1186/s40478-024-01833-9 (PMC11318126; doi:10.1186/s40478-024-01833-9)
Supplement: Supplementary file 5 — Additional file 5. [file 40478_2024_1833_MOESM5_ESM.docx]

**Additional file 5: Arm-level copy number alterations by chordoma outcome**


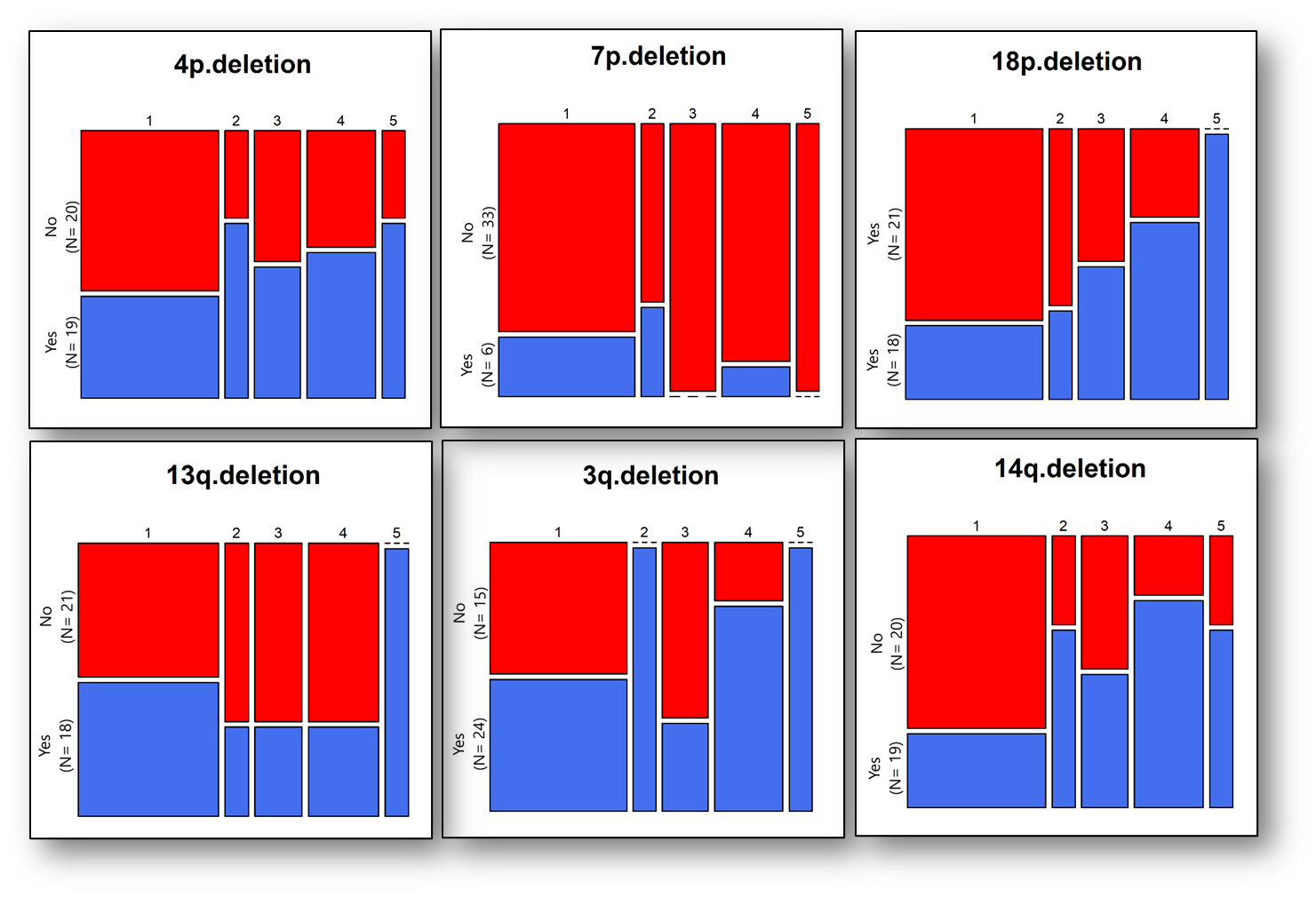


1= Having no chordoma anywhere (N=18).

2= Having chordoma at original site but tumor not growing (N= 3).

3= Having chordoma at the original site or elsewhere and the tumor was growing (N= 6).

4= Having chordoma spreading at other sites, including 12 deaths (N= 9).

5= Died (N= 3).
